# Evaluation of Co‐Developed Strategies to Support Staff of a Mental Health Community Managed Organisation Implement Preventive Care: A Pilot Controlled Trial

**DOI:** 10.1002/hpja.70018

**Published:** 2025-02-25

**Authors:** Casey Regan, Kate Bartlem, Jenna Hollis, Julia Dray, Caitlin Fehily, Elizabeth Campbell, Sarah Leask, Lucy Leigh, Mark Orr, Sumathi Govindasamy, Jenny Bowman

**Affiliations:** ^1^ School of Psychological Sciences, College of Engineering, Science and Environment The University of Newcastle Callaghan New South Wales Australia; ^2^ Population Health Hunter New England Local Health District Wallsend New South Wales Australia; ^3^ The Australian Preventive Partnership Centre (TAPPC) Sax Institute Ultimo New South Wales Australia; ^4^ Population Health Research Program Hunter Medical Research Institute New Lambton Heights New South Wales Australia; ^5^ School of Medicine and Public Health University of Newcastle Callaghan New South Wales Australia; ^6^ Sydney, Graduate School of Health, Faculty of Health University of Technology Sydney New South Wales Australia; ^7^ Flourish Australia Sydney New South Wales Australia

## Abstract

**Background:**

Mental health community managed organisations (CMOs) are well placed to provide preventive care, including behaviour change conversations to address smoking, nutrition, alcohol and physical activity (*snap*). This study evaluates the impact of co‐developed preventive care implementation support strategies, including Healthy Conversation Skills (HCS) training on CMO staff attitudes and perceptions relating to preventive care for *snap* behaviours.

**Methods:**

A non‐randomised controlled pilot trial was undertaken (October 2021–May 2022) with two branches of a mental health CMO (*n* = 1 target; *n* = 1 control) in NSW, Australia. Target group staff received a three‐month implementation support package co‐developed by staff and researchers, including HCS training and educational materials. Staff from both groups completed an online survey at baseline and follow‐up, reporting barriers and facilitators and perceived individual and organisational ability to provide preventive care for each behaviour. Pre and post HCS training, target staff completed surveys reporting barriers and facilitators to having behaviour change conversations, and competency of using ‘open discovery questions’ (a key HCS skill).

**Results:**

Baseline (*n* = 27) and follow‐up (*n* = 17) surveys showed mean scores increased for the target group and decreased for the control group for *n* = 4/8 barrier and facilitator outcomes, and *n* = 7/8 perceived individual and organisational ability of providing care outcomes. Sixteen target group staff participated in HCS training and surveys, with scores improving for skills (*p* = 0.0009), beliefs about capabilities (*p* = 0.0035), intentions (*p* = 0.0283), participant confidence (*p* = 0.0043), perceived usefulness (*p* = 0.004), and competence in using open discovery questions (*p* < 0.0001).

**Conclusions:**

This pilot trial demonstrates the feasibility and potential effectiveness of a co‐developed implementation support package at increasing mental health CMO staff capacity to provide preventive care for multiple health behaviours.

**So What?:**

This evidence can inform future research trials and health policy aimed at supporting CMO staff in delivering comprehensive preventive care.

## 1 Introduction

People with a mental health condition have a reduced life expectancy of up to 30 years compared to people without a mental health condition [1]. This is primarily due to an increased risk of chronic disease, contributed to by a higher prevalence of multiple health behaviours including tobacco smoking, poor nutrition, harmful alcohol consumption, and insufficient physical activity (*snap*) [2, 3]. Research shows this population group are interested in improving their health behaviours and would like to receive support to do so [4, 5], however face material barriers such as low income, lack of access to transport, and insecure housing [6]. Such support, referred to as preventive care [7], is recommended to be routinely delivered in mental health service settings [8]. The ask‐advise‐refer (AAR) is an evidence‐based model of preventive care [9] with demonstrated effectiveness at initiating behaviour change [10]. Preventive care using the AAR framework can be delivered through behaviour change conversations, in which behaviour change is discussed to support an individual to identify actions or solutions to improve their health behaviours [11].

Community managed organisations (CMOs) have been identified as an important setting to deliver preventive care to people with a mental health condition [12]. CMOs (also referred to as non‐government and third sector) provide services to people experiencing various forms of disadvantage, such as mental illness, disability, alcohol and other drug dependence, homelessness, and financial hardship. Mental health CMOs deliver recovery‐oriented services that aim to address holistic needs of people with a mental health condition to improve overall wellbeing such as daily living skills, transport, attending healthcare appointments, employment, education and accommodation and housing [13]. In Australia, the role that CMOs are playing in the mental health sector is increasing, with funding for mental health psychosocial support services growing 10‐fold over the past 25 years [14]. Within the state of New South Wales (NSW), Australia, CMOs employ one quarter of the mental health workforce, and within mental health CMOs 55% are mental health support workers and 14% peer workers [15]. Contact between CMOs and people accessing services is generally at least once or multiple times a week [16, 17], providing CMO staff with opportunities to provide frequent, ongoing preventive care.

Research investigating the levels of preventive care provided by mental health CMOs demonstrates variable levels, indicating support for behaviour change is not being provided consistently [18, 19, 20, 21, 22]. Two studies have investigated the frequency with which staff in Australian CMOs deliver preventive care using the AAR framework for *snap* behaviours [17, 18]. These studies assessed the proportion of staff who reported delivering AAR to at least 80% of the individuals they support. The delivery rates varied depending on the behaviour and the element of AAR: for asking, rates ranged from 42% for alcohol use to 58% for smoking; for advising, rates ranged from 37% for alcohol use to 58% for physical activity; and for referring, rates ranged from 17% for smoking to 31% for physical activity [18, 19]. Studies exploring perspectives of Australian CMO staff report several barriers and facilitators to preventive care delivery, including low CMO staff confidence to provide preventive care, limited perceived uptake of referrals by consumers [19], and staff expressing limited ability to influence behaviour change among consumers [17]. The research emphasises the need to explore strategies for enhancing preventive care delivery in these settings.

Evaluations of interventions to enhance CMO staff delivery of preventive care are limited in both number and rigour. A previous rapid review which evaluating evidence on the delivery of preventive and physical health interventions in mental health CMO settings identified 29 relevant studies published in scientific and grey literature [23]. Eight of these studies evaluated models or initiatives for improving the delivery of physical health care (including but not limited to preventive healthcare), with five interventions demonstrating significant improvements in care receipt or provision or consumer physical health outcomes. All five interventions (three RCT and two pre‐post study designs) included implementation strategies to support integration into practice, such as staff training and information and resource provision. Cochrane systematic review evidence supports the efficacy of training and education in supporting healthcare staff [24], such as through educational meetings and outreach [25] or the distribution of educational materials, resources, and practice guidelines [26], in improving the delivery of care.

Authors of the previous rapid review also noted the importance of using a co‐development approach when designing and delivering preventive care interventions and implementation strategies, to allow relevant barriers to be identified and addressed [23]. Involving staff in the development of implementation support strategies is a participatory approach to health research recommended by mental health organisations (e.g., Mental Health Coordinating Council [13]). This approach recommends that end users play at least an equal role in decision making [27]. This is important to ensure strategies developed are feasible and fit to barriers and enablers within the setting context. The review highlighted a need for studies to develop and explore effective ways for CMOs to implement preventive and physical healthcare interventions, particularly using a co‐development approach [23].

The present study is a pilot implementation trial, which aims to address the paucity of controlled trials exploring the impact of co‐developed strategies to support staff of mental health CMOs implement preventive care for *snap* behaviours [28]. Using a controlled design, this study aimed to evaluate the impact of a co‐developed preventive care (AAR) implementation support package for CMO staff on staff attitudes and perceptions relating to preventive care for *snap*. Additionally, this study aimed to evaluate the immediate post‐training impact of the HCS training on barriers and facilitators to having behaviour change conversations, and staff perceived competence, confidence, importance and usefulness of having behaviour change conversations.

## 2 Methods

### 2.1 Study Design and Setting

A non‐randomised controlled trial was undertaken with two branches of a national non‐for‐profit CMO specialising in mental health in NSW, Australia (see Figure S1). The organisation employs peer workers, mental health workers, team coordinators and managers, and provides community living support (CLS) to assist people living in the community with a diagnosed mental health condition. CLS services include psychosocial supports (i.e., assistance with daily living skills such as cleaning and cooking) that are informed by an individual care plan designed collaboratively between consumers, their family and/or carers, and CMO staff. Staff are expected to embed preventive care relating to physical health and wellbeing conversations to encourage people to maintain a responsive approach toward their physical health, identify and self‐manage their physical health, and access primary health care services (e.g., General Practitioners) to address health concerns. Of the two branches, the branch in closest geographical proximity to the research team was allocated to be the target group for the pilot trial and participated in co‐development workshops [28]. The other branch participated as the control group. Approximately 60 staff are employed across the two branches (target *n* = 20 and control *n* = 40). The target group consisted of two sites, both managed by the one manager, and received implementation support strategies over 3 months to assist them to deliver preventive care (December 2021 to March 2022). The support strategies were co‐developed with staff and management prior to the trial as described in Regan et al. (2023) [28]; several staff who participated in the co‐development workshops were from the target site. All staff in both the target and control group were invited to participate in online surveys at baseline (October 2021) and follow‐up (May 2022), and target group staff participating in training were invited to participate in pre‐post training surveys.

### 2.2 Model of Care

The strategies aimed to support staff to deliver a simplified model of preventive care (‘Ask, Advise, Refer’ [AAR]) to consumers to support health behaviour change for: tobacco smoking, nutrition, alcohol consumption and physical activity (*snap*). Based on learnings from the co‐development workshops [28], the terminology of the AAR model was reframed to align with language preferences of staff and to be consistent with CMO practice. Hence, the model of care was communicated to staff as ‘CAC’: ‘conversations’ (have conversations about healthy behaviours and behaviour change); ‘advice’ (provide brief advice on how to improve their behaviours to meet the Australian national guidelines (e.g., engaging in at least 150 min of physical activity per week) [29], and the benefits of doing so); and ‘connections’ (connect consumers to referral services that specialise in behaviour change support e.g., evidence‐based, state‐wide telephone support services such as the NSW Quitline for smoking [30] and the NSW Get Healthy Information and Coaching Service for nutrition, inadequate physical activity and alcohol consumption) [31].

### 2.3 Implementation Support

The support strategies were co‐developed with CMO management and staff to align with barriers and facilitators impacting preventive care delivery, with the co‐development methods previously published [28]. Two evidence‐based implementation support strategies [32, 33] were delivered over 3 months: (1) enabling resources and prompts including a step‐by‐step and referral guide for each *snap* behaviour (also referred to as educational materials [34]) (December 2021); and (2) Healthy Conversation Skills (HCS) training (also referred to as educational meetings [34]) (March 2022). Resources (a step‐by‐step and a referral guide) were also provided for poor sleep hygiene concurrently with other resources at the request of the organisation, however these were not part of the evaluation.

#### 2.3.1 Step‐By‐Step and Referral Connections Guides (Educational Materials)

A step‐by‐step guide to having conversations, providing brief advice and connecting to referral services for each *snap* behaviour was produced by the research team. Four step‐by‐step guides (one for each behaviour) were developed, each A4 in size and double‐sided. The guides detailed: examples of conversation starters, open discovery questions, and strengths‐based language quotes; recommended Australian national behaviour guidelines; risk assessment tools (e.g., AUDIT‐C for the alcohol consumption guide); physical, mental and financial benefits of healthy changes; practical tips on how to change; and referral services that specialise in behaviour change for the specific behaviour. Forty hardcopy booklets were provided to the target group in December 2021.

A connections guide which detailed a range of referral services for smoking cessation, alcohol reduction, increasing physical activity and improving healthy eating was also developed. Information about the referral service including the name, what it provides, where it is located, how much it costs, who is eligible to be referred, and how to connect consumers to the service (e.g., phone, website), was included. The guide was 12 A4 single‐sided pages, and 40 hardcopy guides were provided to the target group in December 2021. The step‐by‐step guides and connections guides were also stored electronically in an online Microsoft Teams group share folder to which all staff from the target group had access.

#### 2.3.2 Healthy Conversation Skills (HCS) Training (Educational Meetings)

HCS training is an evidenced‐based program to support health workers to engage in useful behaviour change conversations with consumers [35] (Figure S2). The program trains health workers to use a person‐centred and strengths‐based approach to encourage consumers to set goals and identify their own solutions to improve their health behaviours [35]. Staff from a range of health professions who have participated in HCS training have demonstrated improved confidence and competence in having behaviour change conversations [35, 36], as well as reduced barriers to supporting consumers with behaviour change [37]. HCS is based on Social Cognitive Theory [38], and training delivery is informed by the taxonomy of behaviour change techniques [39], using an interactive and participatory approach to learning. During the training, the trainer provides a demonstration of HCS as well as opportunities for participants to practice their skills and to identify discrepancies between their current and desired communication style. For example, a key HCS is responding with open discovery questions (open‐ended questions that begin with ‘how’ and ‘what’). There is no use of technology and participants are discouraged from taking notes and are encouraged to fully engage with the experiential style of training.

HCS training usually consists of two half day (3–4 h) training sessions (session 1 and 2) conducted 1–2 weeks apart to enable participants time to practice their skills and reflect between sessions. For pragmatic reasons, to meet organisational needs regarding capacity to release staff to attend training, HCS training (lite) which involves session one only (3 h) was conducted in March 2022 (one training session at each of the two sites of the target group). This was approximately 5 months after baseline data collection and 2 months prior to follow‐up data collection.

### 2.4 Control Group

The control group staff received their usual guidance in line with their service expectations and continued to provide their usual support services to clients. They were not involved in co‐development, and did not receive any support strategies delivered as part of the pilot trial.

### 2.5 Participants and Recruitment

All target group staff were given the opportunity to receive the support strategies (including the HCS training), and all target group staff who participated in the HCS training were eligible to participate in the HCS surveys at the start and end of the 3 h training session. All staff from both groups were invited to participate in online staff surveys at baseline and follow‐up.

#### 2.5.1 Online Staff Survey

All staff from both target and control groups who were = > 18 years old and provided support to people with a mental health condition were eligible to participate. At both baseline and follow‐up, the manager from each group sent their respective staff an email with an information statement and a link to the anonymous survey. A reminder email was sent after 2 weeks.

#### 2.5.2 HCS Training Surveys

The manager of the target group invited (via email) all staff to attend the training. Information statements regarding the HCS survey data collection were emailed to participants prior to attending training and provided again at the beginning of each survey, and consent was implied by completing each survey. Participation in the HCS surveys was voluntary and anonymous, and staff were able to attend the training but choose not to participate in the data collection.

### 2.6 Data Collection and Measures

#### 2.6.1 Online Staff Surveys

Outcome data to explore the impact of the co‐developed support strategies were collected through online survey at two time points using REDCap [40]. The baseline survey was conducted prior to delivery of the support strategies (October 2021), and the follow‐up survey was conducted after delivery of the support strategies (May 2022), 8–10 weeks after the HCS training and 4 months after delivery of the step by step and referral guides (Figure S1). The online surveys were open for 2 weeks each, and the following items were asked at both timepoints.

##### 2.6.1.1 Participant Characteristics

Participants were asked to identify the branch they were employed at, age, Aboriginal and Torres Strait Islander origin identity, education level, qualification, employment type, length of employment and role with the organisation.

##### 2.6.1.2 Perceptions of Routinely Providing Preventive Care

Participants were asked to indicate on a 5‐point likert scale (1 = completely disagree; 2 = disagree; 3 = neither agree nor disagree; 4 = agree; 5 = completely agree) to what extent they agree or disagree with statements about routinely providing preventive care for *snap* behaviours. All items were designed by the research team and were informed by previous research conducted with mental health staff working in CMO to assess perceptions (including barriers and facilitators) toward preventive care delivery [16, 17, 19]. Perceptions of routinely providing preventive care were measured through 16 items across three categories: barriers and facilitators to providing preventive care (*n* = 8); perceived individual ability of providing preventive care (by *snap*) (*n* = 4); and perceived organisational ability of providing preventive care (by *snap*) (*n* = 4) (Table 1). Items included within the barriers and facilitators to providing preventive care category (*n* = 8) were based on the Theoretical Domains Framework (TDF) domains: skills, knowledge, social influences, beliefs about capabilities, beliefs about consequences and environmental context and resources (Table 1). The TDF is an integrative and validated framework that enables the mapping of barriers and facilitators to 12 specific behavioural domains [41, 42]. The specified domains were expected to be important in the current setting as they were identified as barriers to preventive care delivery during the co‐development workshop [28] and in previous studies [17, 19]. The other two categories regarding perceived individual and organisation ability included questions that asked specifically about each *snap* behaviour, as previous studies suggest variability in preventive care provision across behaviours [18]. A response option of ‘prefer not to say’ was available for all items.

**TABLE 1:** Items in online staff survey assessing perceptions of routinely providing preventive care (three categories) and items in HCS training survey (three categories).

Topic	Items	Scale
Online staff survey		
Barriers and facilitators to providing preventive care	(1) I feel confident to routinely provide preventive care for health behaviours (beliefs about capabilities)	5‐point Likert scale
	(2) I have the skills and knowledge to routinely provide preventive care for health behaviours (knowledge and skills)	
	(3) I have the time to routinely provide preventive care for health behaviours (environmental context and resources)	
	(4) I have adequate resources to routinely provide preventive care for health behaviours (environmental context and resources)	
	(5) I have adequate support from my organisation/manager to routinely provide preventive care for health behaviours (environmental context and resources)	
	(6) It is important to routinely provide preventive care for health behaviours (beliefs about consequences)	
	(7) The people I support want me to provide preventive care for their health behaviours (social influences)	
	(8) Routinely providing preventive care could benefit the mental health of the people we support (beliefs about consequences)	
Individual ability to provide preventive care (by *snap*)	(1) I feel able to routinely provide preventive care for smoking	5‐point Likert scale
	(2) I feel able to routinely provide preventive care for alcohol	
	(3) I feel able to routinely provide preventive care for physical activity	
	(4) I feel able to routinely provide preventive care for nutrition	
Organisational ability to provide preventive care (by *snap*)	(1) My team at Flourish is able to routinely provide preventive care for smoking	5‐point Likert scale
	(2) My team at Flourish is able to routinely provide preventive care for alcohol	
	(3) My team at Flourish is able to routinely provide preventive care for physical activity	
	(4) My team at Flourish is able to routinely provide preventive care for nutrition	
HCS training survey		
Barriers and facilitators to having BCC^a^	(1) I have been trained how to have behaviour change conversations in routine consultations with individuals/clients (skills domain)	7‐point Likert scale^c^
	(2) I have the skills to have behaviour change conversations in routine consultations with individuals/clients (skills domain)	
	(3) I have practiced having behaviour change conversations in routine consultations with individuals/clients (skills domain)	
	(4) I am confident that I can have behaviour change conversations in routine consultations with individuals/clients even when individuals/clients are not motivated (beliefs about capabilities domain)	
	(5) I am confident that I can have behaviour change conversations in routine consultations with individuals/clients even when there is little time (beliefs about capabilities domain)	
	(6) I am confident that if I wanted I could have behaviour change conversations in routine consultations with individuals/clients (beliefs about capabilities domain)	
	(7) I will definitely have behaviour change conversations in consultations with individuals/clients in the next 3 months (intentions domain)	
	(8) I intend to have behaviour change conversations in consultations with individuals/clients in the next 3 months (intentions domain)	
	(9) For how many of your next 10 clients/individuals do you intend to have behaviour change conversations in consultations? (intentions domain)^b^	
	(10) How strong is your intention to have behaviour change conversations with individuals/clients in consultations in the next 3 months? (intentions domain)	
Confidence, importance, and usefulness to having BCC^a^	(1) On a scale of 1–10 how confident do you feel about supporting clients/individuals to make a behaviour change?	10‐point Likert scale
	(2) On a scale of 1–10, how important is it for you to support clients/individuals to make a behaviour change?	
	(3) On a scale of 1–10, how useful do you think the conversations you have are at supporting individuals to make a behaviour change?	
Competency of using open discovery question responses	(1) “I need to lose weight, but I don't like vegetables.” You say:	Open
	(2) “I should cut down on my alcohol intake, but my partner likes to open a bottle of wine after work.” You say:	
	(3) “I just do not seem to have time to do any exercise.” You say:	
	(4) “I have lost count of the number of times I have tried to stop smoking—it is hopeless!” You say:	

^a^ Behaviour change conversations.

^b^ 10‐point Likert scale.

^c^ 1 = strongly disagree, 2 = disagree, 3 = slightly disagree, 4 = neutral, 5 = slightly agree, 6 = agree, 7 = strongly agree.

##### 2.6.1.3 Recall of Pilot Implementation Support Strategies

In the follow‐up survey, participants were asked if they were aware of strategies implemented in their service to support provision of preventive care. Participants were also asked if they attended HCS training.

#### 2.6.2 HCS Training Surveys

Evaluation data for the HCS training were collected at two time points. The pre‐ and post‐training surveys were conducted immediately prior to and at the conclusion of training to maximise participant response rates and increase the validity of the measures by minimising recall bias and the influence of confounding factors (e.g., discussions with colleagues, and engaging in additional training). Participants' pre and post responses were not matched.

##### 2.6.2.1 Participant Characteristics (Pre‐Training Only)

Including participant gender, Aboriginal or Torres Strait Islander origin, location of training (site), professional qualification, length of employment and employment status were collected in the pre‐training survey.

##### 2.6.2.2 Confidence, Importance and Usefulness (Pre‐ and Post‐Training)

Three questions assessed perceived confidence, importance and usefulness of having behaviour change conversations with consumers. Responses were reported on a 10‐point Likert scale, where one was the lowest score (not confident/important/useful) and 10 was the highest (very confident/important/useful). The measure of importance was included to gauge the priority CMO staff give to behaviour change conversations with consumers as one of many competing priorities, and to determine their level of receptivity to training in skills that support behaviour change. The items are presented in Table 1.

##### 2.6.2.3 Barriers and Facilitators to Having Behaviour Change Conversations (Pre‐ and postP‐Training)

Survey items assessing potential barriers to having behaviour change conversations were developed from the TDF [41, 42]. Three of the domains of the TDF survey developed by Huijg et al. [43, 44] were included based on identified barriers to having behaviour change conversations among CMO staff highlighted in Regan et al. (2023) [28]. Ten TDF survey items assessed perceived barriers and facilitators: skills (*n* = 3 items); beliefs about capabilities (*n* = 3); and intentions (*n* = 4) (Table 1). For one TDF survey item participants reported the score out of 10 (intentions domain: For how many of the next 10 people you support do you intend to have behaviour change conversations in consultations?).

##### 2.6.2.4 Competency in HCS (Pre‐ and Post‐Training)

To assess competency in using ‘open discovery questions’ (a key healthy conversation skill), participants were provided with four written statements made by hypothetical consumers about difficulties with changing each *snap* behaviour (see Table 1 for statements). Participants were asked to provide individual written responses to these statements mimicking what their verbal response would be if a consumer made this comment to them whilst they were providing care. Participant responses to the four statements were coded into one of seven possible response categories by a researcher with expertise in HCS (JLH) using an existing coding matrix used in previous studies [35, 37, 45] (see Table S3).

### 2.7 Statistical Analysis

All statistical analyses were programmed using SAS v9.4 (SAS Institute, Cary, North Carolina, USA). The samples for each survey were not matched.

#### 2.7.1 Online Staff Surveys

##### 2.7.1.1 Participant Characteristics (Baseline and Follow‐Up)

Descriptive statistics (frequencies and proportions) were produced for participant characteristics of the control and target groups by timepoint.

##### 2.7.1.2 Perceptions of Routinely Providing Preventive Care (Baseline and Follow‐Up)

Descriptive statistics (mean, SD) were produced for barriers and facilitators to providing preventive care; perceived individual ability of providing preventive care; and perceived organisational ability of providing preventive care of the target and control groups, stratified by baseline and follow‐up. Change in mean scores from baseline to follow‐up were compared for each group.

#### 2.7.2 HCS Training Surveys

##### 2.7.2.1 Participant Characteristics (Pre‐Training Only)

Descriptive statistics (frequencies and proportions) were produced.

##### 2.7.2.2 Confidence, Importance and Usefulness (Pre‐ and Post‐Training)

Descriptive statistics (median, Q1 & Q3) were produced for the three outcomes (perceived confidence, importance and usefulness) related to attitudes toward having behaviour change conversations, stratified by pre‐ and post‐training. Kruskal–Wallis H tests were performed to investigate if a statistically significant change occurred in results pre‐ versus post‐training for each outcome. Statistical significance was assessed at the 5% level.

##### 2.7.2.3 Barriers and Facilitators to Behaviour Change Conversations (pre‐ and Post‐Training)

Descriptive statistics (median, Q1 & Q3) were produced for the three TDF domains (skills, beliefs about capabilities and intentions) associated with barriers or facilitators to having behaviour change conversations, stratified by pre‐ and post‐training. Median values were calculated for each TDF domain by summing the scores for each item within the domain and dividing by the total number of items. TDF items that were worded negatively were inverted before being added to the composite totals. The one TDF survey item participants reported the score out of 10 (intentions domain) was scaled to a maximum score of seven for the analysis. Kruskal–Wallis H tests were performed to investigate if a statistically significant change occurred in results pre‐ versus post‐training for each domain median. Statistical significance was assessed at the 5% level.

##### 2.7.2.4 Open Discovery Questions (Pre‐ and Post‐Training)

Descriptive statistics were used to compare the frequency and proportion of participant responses that use open discovery questions (Table S3, code 7) for each behaviour and all behaviours combined (‘total’) for pre‐ and post‐training. A Fisher's Exact test was performed to determine if a statistically significant change in use of open discovery question responses (code 7) occurred pre‐ versus post‐training (Table 6).

## 3 Results

### 3.1 Sample Characteristics

There were 27 completed surveys at baseline (*n* = 14/20 target staff (70%) and *n* = 13/46 control (28%)) and 17 at follow‐up (*n* = 10/20 target staff (50%) and *n* = 7/40 control (18%)). Table 2 displays sample characteristics of the target and control groups by timepoint. The majority of staff reported a TAFE or University degree education level, professional qualifications in support work, employment with the CMO for longer than 12 months, full time employment and predominantly worked in a person‐centred support role (Table 2). Of the 13 target group staff completing the follow‐up survey, 5 recalled being aware of the implementation support strategies (38.5%), and 5 recalled attending the HCS training (38.5%). Sixteen staff from the target group (80% of total staff, *n* = 20) participated in the HCS training (11 at site A and 5 at site B) and all 16 participated in the pre‐ and post‐ training surveys (Table 3). The majority of staff reported professional qualifications in support work, employment with the CMO for longer than 12 months (with half longer than 5 years), and full‐time employment (Table 3).

**TABLE 2:** Characteristics of participants from the control and target groups who undertook the pilot trial baseline and follow‐up surveys.

Variable	Level	Control				Target			
		Baseline (*N* = 13)		Follow‐up (*N* = 7)		Baseline (*N* = 14)		Follow‐up (*N* = 10)	
		*N*	%	*N*	%	*N*	%	*N*	%
Age	Mean (SD)	42.1	12.3	39.1	10.2	50.1	15.3	53.3	14.5
	Median (min, max)	41.0	(23, 63)	39.0	(29, 61)	57.0	(27, 71)	59.0	(23, 72)
Aboriginal	Yes	3	23.1	1	14.3	0		0	
Education	Less than Year 10	1	7.7	0		0		0	
	Year 10 (High School Certificate)	1	7.7	0		1	7.1	1	10.0
	TAFE^a^	9	69.2	4	57.1	9	64.3	5	50.0
	University (Degree or higher)	2	15.4	2	28.6	4	28.6	3	30.0
Qualification	Support work^b^	10	76.9	4	57.1	10	71.4	8	80.0
	Allied health^c^	0		1	14.3	1	7.1	1	10.0
	Other	2	15.4	1	14.3	2	14.3	1	10.0
	Prefer not to say	1	7.7	1	14.3	1	7.1	0	
Length of employment	4 months or less	1	7.7	0		0		1	10.0
	Between 5 and 12 months	3	23.1	0		1	7.1	1	10.0
	More than 12 months	9	69.2	7	100.0	13	92.9	7	70.0
Type of employment	Full time	10	76.9	5	71.4	10	71.4	4	40.0
	Part time	3	23.1	2	28.6	3	21.4	5	50.0
	Casual	0		0		1	7.1	1	10.0
Role	Administrative/Managerial	1	7.7	3	42.9	3	21.4	1	10.0
	Person‐centred support	9	69.2	4	57.1	10	71.4	8	80.0
	Other	3	23.1	0		1	7.1	1	10.0

^a^ Certificate, diploma, advanced diploma.

^b^ Including peer, youth or mental health work.

^c^ Including nursing, psychology, or occupational therapy.

**TABLE 3:** Characteristics of HCS training participants (target group).

Characteristic	Level	*N*	%
Site	Site 1	5	31.3
	Site 2	10	62.5
	Both site 1 and 2	1	6.3
Gender	Female	10	62.5
	Male	6	37.5
Qualification	Support work	13	81.3
	Allied health	1	6.3
	Other^a^	2	12.5
Length of employment	4 months or less	2	12.5
	Between 5 and 12 months	3	18.8
	1–4 years	3	18.8
	5+ years	8	50.0
Type of employment	Full time	10	62.5
	Part time	6	37.5

### 3.2 Online Staff Survey Outcomes: Impact of Support Strategies

Descriptive statistics for each of the 16 outcomes assessed in the baseline and follow‐up staff surveys (barriers and facilitators to providing preventive care *n* = 8; perceived individual ability of providing preventive care (by *snap*) *n* = 4; and perceived organisational ability of providing preventive care (by *snap*) *n* = 4) are displayed by control and target group by timepoint in Table 4. Mean scores increased for the target group and decreased for the control group for four of the eight barrier and facilitator outcomes about routinely providing preventive care for health behaviours (items 1, 3, 5, 6). For example, there was an increase in mean scores from baseline to follow‐up in the target group for item 3 (‘I have the time…’) of +0.34 (vs control −0.26). Mean scores increased for the target group and decreased for the control group for five of eight outcomes relating to perceived individual and organisational ability of providing care for each behaviour. For example, regarding perceived *individual* ability to provide preventive care, there was an increase in mean scores from baseline to follow‐up in the target group for nutrition of +0.20 (vs control −0.03), and physical activity of +0.15 (vs control −0.03). Regarding perceived *organisation* ability, there was an increase in mean scores from baseline to follow‐up in the target group for smoking of +0.24 (vs control −0.03), and nutrition of +0.20 (vs control −0.17).

**TABLE 4:** Staff survey outcomes.

Variable		Control			Target		
		Baseline (*N* = 15)	Follow‐up (*N* = 8)	Mean change	Baseline (*N* = 17)	Follow‐up (*N* = 13)	Mean change
Barriers and facilitators to providing preventive care							
(1) I feel confident…^a^		4.47 (0.52)	4.29 (0.49)	−0.18	3.88 (1.05)	4.00 (0.82)	+0.12
(2) I have the skills and knowledge…^a^		4.33 (0.72)	4.29 (0.49)	−0.04	3.94 (0.97)	3.92 (0.86)	−0.02
(3) I have the time…^a^		3.93 (0.88)	3.57 (1.13)	−0.26	3.35 (1.17)	3.69 (0.85)	+0.34
(4) I have adequate resources…^a^		4.00 (1.20)	4.00 (1.00)	−0.00	3.59 (1.12)	3.54 (1.05)	−0.05
(5) I have adequate support from my organisation/manager…^a^		4.27 (0.70)	4.14 (0.38)	−0.13	3.88 (0.99)	3.92 (0.95)	+0.04
(6) It is important…^a^		4.73 (0.46)	4.71 (0.49)	−0.02	4.18 (0.95)	4.46 (0.52)	+0.28
(7) The people I support want me…^a^		3.87 (0.92)	3.57 (0.53)	−0.20	3.06 (0.97)	2.62 (0.87)	−0.44
(8) Routinely providing preventive care could benefit the mental health of the people we support		4.60 (0.63)	4.71 (0.49)	+0.11	4.47 (1.01)	4.54 (0.52)	+0.07
Individual ability of providing preventive care (by *snap*)							
^b^…Smoking		4.33 (0.82)	4.38 (0.52)	+0.05	3.76 (1.09)	4.00 (0.82)	+0.24
^b^…Nutrition		4.53 (0.52)	4.50 (0.53)	−0.03	3.88 (1.11)	4.08 (0.86)	+0.20
^b^…Alcohol		4.20 (1.01)	4.38 (0.52)	+0.18	3.65 (1.11)	4.00 (0.71)	+0.35
^b^…Physical activity		4.53 (0.52)	4.50 (0.53)	−0.03	4.00 (1.12)	4.15 (0.80)	+0.15
Organisational ability of providing preventive care (by *snap*)							
^c^…Smoking		4.47 (0.92)	4.44 (0.53)	−0.03	4.18 (1.13)	4.23 (0.93)	+0.24
^c^…Nutrition		4.73 (0.46)	4.56 (0.53)	−0.17	4.18 (1.13)	4.23 (0.93)	+0.20
^c^…Alcohol		4.33 (1.05)	4.33 (0.50)	−0.00	4.06 (1.14)	4.15 (0.90)	+0.35
^c^…Physical activity		4.67 (0.49)	4.44 (0.53)	−0.23	4.29 (1.16)	4.15 (1.07)	−0.03

^a^ … To routinely provide preventive care for health behaviours.

^b^ I feel able to routinely provide preventive care for….

^c^ My team at the CMO is able to routinely provide preventive care for….

* 5 point Likert scale where 1 = strongly disagree; 2 = disagree; 3 = neither disagree or agree; 4 = agree; 5 = strongly agree.

### 3.3 HCS Training Outcomes: Impact of the Training Support Strategy

#### 3.3.1 Barriers and Facilitators to Having Behaviour Change Conversations

From pre‐training to post‐training, there were significant increases in all three of the TDF domain scores with improvements in participant's self‐reported skills (median 5.3 to 6.3; *p* = 0.0009), beliefs about capabilities (median 5.5 to 6.3; *p* = 0.0035), and intentions (median 5.7 to 6.5; *p* = 0.0283) of having behaviour change conversations (score out of 7, 7 is high) (Table 5).

**TABLE 5:** Participant perceptions toward having behaviour change conversations (BCC) before and after HCS training (median (Q1, Q3)).

Outcome		Pre‐training (*N* = 16)	Post‐training (*N* = 16)	*p*
Barriers and facilitators to having BCC				
Skills		5.33 (4.67, 6.00)	6.33 (6.00, 7.00)	0.0009*
Beliefs about capabilities		5.50 (4.67, 6.00)	6.33 (5.83, 6.83)	0.0035*
Intentions		5.71 (5.29, 6.25)	6.46 (5.83, 6.92)	0.0283*
Confidence, importance, and usefulness of having BCC				
Confidence		7.50 (7.00, 8.00)	9.00 (8.00, 10.00)	0.0043*
Importance		8.50 (7.50, 9.00)	9.00 (8.00, 10.00)	0.2632
Usefulness (value)		7.50 (7.00, 9.00)	9.00 (9.00, 10.00)	0.0040*

* Statistically significant *p*‐value.

#### 3.3.2 Confidence, Importance and Usefulness of Having Behaviour Change Conversations

From pre‐training to post‐training, there were significant increases in participants' self‐reported confidence (median 7.5 to 9.0; *p* = 0.0043) and usefulness/value (median 7.5 to 9.0, *p* = 0.004), but not importance (median 8.5 to 9.0; *p* = 0.2632), of having behaviour change conversations (score out of 10, 10 is high) (Table 5).

#### 3.3.3 Open Discovery Questions

From pre‐training to post‐training, significant improvements in using open discovery questions were observed for all four behaviour statements: smoking (0% to 92%; *p* < 0.0001), nutrition (23% to 77%; *p* = 0.0169), alcohol (8.3% to 100%; *p* < 0.0001), and physical activity (7.7% to 100%; *p* < 0.0001); and for all behaviours combined (9.6% to 89.4%; *p* < 0.0001) (Table 6; Figure S4).

**TABLE 6:** The impact of HCS training on participants use of Open Discovery Style responses (a key HCS skill) for each behaviour.

Behaviour	Pre‐training (*N* = 16)		Post‐training (*N* = 16)		*p*
	*N*	%	*N*	%	
Nutrition	3/13	23.1	10/13	76.9	0.0169*
Alcohol	1/12	8.3	10/10	100.0	< 0.0001*
Smoking	0/14	0.0	10/12	83.3	< 0.0001*
Physical activity	1/13	7.7	12/12	100.0	< 0.0001*
All behaviours combined	5/52	9.6	42/47	89.4	< 0.0001*

* Statistically significant *p*‐value.

## 4 Discussion

This pilot implementation trial was the first to evaluate the impact of co‐developed support strategies for mental health CMO staff on their attitudes and perceptions on providing preventive care for *snap*. The trial demonstrated improvements in the target group relative to the control group in four of eight outcomes relating to perceived care barriers and facilitators including confidence, time, managerial support and importance. There were also relative improvements in seven of eight outcomes relating to perceived individual and organisational ability of providing preventive care for all *snap* behaviours, except for organisational ability to provide care for physical activity. When examining the impact specifically of one of the implementation support strategies, the HCS training program, results demonstrated significant improvements in five of six outcomes, including competence, skills, beliefs about capabilities, intentions, confidence and usefulness (value) of having behaviour change conversations immediately post training. These findings suggest that implementation strategies co‐developed with CMO staff may address at least some barriers and facilitators to preventive care delivery and may lead to improved perceived ability to provide preventive care individually and organisationally.

This study adds to a limited literature‐base of co‐developed implementation support targeting preventive care delivery for multiple health behaviours that have been conducted in CMO settings [23]. The positive outcomes of this pilot trial may be attributable to the participatory approach used to identify suitable strategies for addressing barriers to preventive care delivery in CMOs, as highlighted during the preceding co‐development process [28] and supported by evidence in previous research [16, 17, 19]. The co‐development process provided an opportunity for staff to discuss and vote for support strategies within the context of barriers in their services, allowing participants to collaborate on what the support strategies would be and how best to implement them. Implementation research in mental health service settings [46], general community health settings [47] and other settings [48] recognise strategies should address identified barriers and facilitators to preventive care relevant to each services to increase the likelihood for optimum effectiveness. This is equally important in mental health CMO settings as different organisations are likely to experience unique barriers and facilitators to preventive care delivery depending on staff, consumers, service type and culture [17, 19, 23]. The results of this pilot trial provide preliminary evidence that the co‐developed support strategies addressed some barriers to preventive care delivery in the CMO setting.

Half of the barrier and facilitator outcomes increased for the target group and decreased for the control group (confidence; time; adequate managerial support and importance). The observed decreases in the control group may be partially attributed to the small sample size at follow‐up (*n* = 8), which increases the likelihood of random fluctuations and variability in outcomes. The support as delivered did not improve some barriers and facilitators outcomes (knowledge and skills; adequate resources and people want support). It is surprising the mean score for adequate resources decreased from baseline to follow‐up, as one of the implementation strategies included educational materials (step‐by‐step and referral connections guides). However, fidelity data indicated that a low proportion of participants who completed the follow‐up survey recalled being aware of the pilot implementation support resources. It is unknown whether this was due to issues with recognition and/or recall, or due to their delivery. These results suggest that additional strategies may be required to specifically address the barriers and facilitators that were not impacted in the current trial. For instance, strategies that aid staff recall and engagement, such as audit and feedback [49], automated reminders [50], and leadership/managerial supervision [51], could enhance the accessibility and prominence of support resources. However, future research should prioritise co‐designing these strategies with stakeholders to ensure they are contextually appropriate for CMOs.

Staff survey results regarding perceived individual and organisational ability across each *snap* behaviour suggested a positive impact of the implementation strategies. Although descriptive, increases in mean scores across all behaviours (except physical activity for organisational ability) for the target group suggest the co‐developed support strategies may have helped improve staff perceived ability to provide preventive care. It is unclear why perceived organisational ability to provide care for physical activity was not impacted, and future research could assess whether additional or different implementation strategies are required to address barriers related to physical activity care. Overall, staff survey results indicate perceived ability did not differ substantially between behaviours, perhaps with exception to physical activity, suggesting support strategies adequately addressed multiple behaviours. Notwithstanding, results are from exploratory, non‐powered analysis and the magnitude of differences is unclear. These findings support the inclusion of strategies to support staff to deliver preventive care for multiple (as opposed to single) health behaviours in the context of preventive care delivery, however more rigorous evaluation of such strategies is required.

To the authors' knowledge, this is the first study of HCS training among a cohort of mental health staff and provides preliminary evidence for the effectiveness of HCS training for mental health CMO staff. To evaluate the training, the current study utilised measures of competence, confidence, importance, usefulness and validated TDF survey items that have been previously used by researchers to evaluate HCS training among other health professionals [35, 36, 37, 42, 43, 45]. This has enabled comparison with previous HCS evaluations and demonstrated the robustness of HCS training in producing desired outcomes across clinical and non‐clinical health profession groups and settings [35, 37, 45]. CMO staff confidence and usefulness of having behaviour change conversations increased post‐training, while perceived importance of having behaviour change conversations did not significantly increase. In the current study, staff reported high levels of importance pre‐ and post‐training, suggesting that staff who attended the training already believe that supporting the people they care for to improve health behaviours is important. This finding suggests that this group of participants are staff who are already motivated to deliver preventive care in their work role. The study adds to the emerging evidence [37] that HCS training addresses three key health care provider barriers to having behaviour change conversations: skills, belief about capabilities and intentions. Such improvements were intended and expected as the training is underpinned by a range of evidence‐based behaviour change techniques and provides multiple opportunities for participants to practise HCS and set goals for future practice. Also consistent with previous HCS evaluations [35, 36, 37, 45], the training was effective in increasing staff competence in using ‘open discovery questions’ in response to hypothetical client behaviour statements. The use of predominately open discovery questions post‐training demonstrates an ability for CMO staff to use a more exploratory, person‐centred approach to having behaviour change conversations that empowers clients to reflect on their behaviour/s and identify a solution for themselves. The results demonstrate the applicability of HCS training for all *snap* behaviours, and given that engaging in behaviour change conversations is a key element of preventive care delivery, many mental health staff report difficulty in doing so [17, 19, 52], thus training staff in HCS could be an efficient capacity‐building approach to support preventive care delivery for different behavioural topics.

## 5 Strengths and Limitations

Claims regarding the effectiveness of strategies are not intended given the pilot trial's small‐scale and descriptive nature. However, results support the feasibility of the co‐development methdology and implementation of the support strategies. The non‐randomisation design is a limitation due to potential selection bias and confounding variables, but it was appropriate for a pilot study focused on feasibility and preliminary insights to inform future, more rigorous randomised trials. Conducting the trial with two branches of a national CMO may limit the generalisability of findings to other CMOs or other community‐based mental health settings. Additionally, the follow‐up survey suggested there was low awareness of the pilot implementation support strategies, raising uncertainty about whether this reflects low reach among all staff in the target group or just the follow‐up sample. The limited change on some outcomes in the online staff survey may be due to low exposure among the sample, with only 5/13 target group survey participants reported participating in the HCS training. Despite these limitations, findings suggest the strategies may address some but not all barriers to delivering preventive care, and supports the value of HCS training as part of an implementation support package, with the HCS training having a positive impact pre‐post when adopted in a mental health CMO setting.

## 6 Future Research

Future studies should look at ways to increase exposure to and use of support strategies to staff and may explore suitable approaches during co‐development to achieve reach. For example, employment of implementation officers to support staff receipt of strategy materials may be beneficial. Challenges in obtaining input from a representative sample of staff to evaluate the impact of the pilot strategies could similarly be addressed during co‐development, in which evaluation tools and methods to facilitate data collection could be designed with staff to ensure they are acceptable and feasible. Researchers should also consider survey measures utilising visual stimulus of the strategies to enhance recognition, as visual aids improve understanding and recall [53]. Future HCS research could identify and test how other post‐training strategies (in addition to HCS training) can support CMO staff to use HCS when delivering routine preventive care. Future trials of HCS training in mental health CMO settings could investigate whether improvements in staff perceptions and barriers to having behaviour change conversations are sustained in the long term, and assess the impact of HCS training on CMO staffs' provision of preventive care for snap behaviours and consumer health behaviour outcomes. Further, future research may consider longer data collection periods to evaluate the sustainability of any effects, particularly in relation to lasting changes in staff behaviour.

Importantly, future trials in this setting should incorporate consumer perspectives using a genuine codesign approach. For instance, many CMOs maintain established consumer advisory groups that could be leveraged, with careful consideration to avoid increasing burden on one repeated group of people. Additionally, service consumers could be engaged pre‐ and post‐trial through surveys, interviews and focus groups and engagement should occur across all research stages from the initial design phase to dissemination.

## 7 Conclusion

In conclusion, the results suggest that the co‐developed implementation strategies piloted in this study to support mental health CMO staff in providing preventive care, addressed relevant barriers and facilitators and improved staff perceived ability to provide care across the *snap* behaviours. Findings in this pilot study suggest the co‐development strategies warrant further development and rigorous testing. This study highlights important learnings about the feasibility and value of HCS training and enabling resources and prompts as potentially effective implementation strategies in a mental health CMO setting. Future research could benefit from investigating the extent to which strategies are delivered; investigating the awareness and use of strategies to improve implementation outcomes; and utilising rigorous randomised trial designs and larger sample sizes to enable statistical inference across all outcomes. Overall, the study highlights the importance of co‐developed implementation strategies to support delivery of care for multiple health behaviours in the context of preventive care interventions in mental health CMO settings.

## Ethics Statement

The trial was approved by the University of Newcastle Human Research Ethics Committees (Approval No. H‐2020‐0435). Informed consent was obtained from all participants involved in the study indicating that they had read and understood the study information statement.

## Consent

The authors have nothing to report.

## Conflicts of Interest

The authors declare no conflicts of interest.

## Supporting information


**Data S1:** Supporting Information.

## Data Availability

Restrictions apply to the availability of these data. The data are not publicly available due to protecting the confidentiality of study participants where data contains easily identifiable information.
