# Supplementary material for: Evaluation of Co‐Developed Strategies to Support Staff of a Mental Health Community Managed Organisation Implement Preventive Care: A Pilot Controlled Trial
Source: Health Promot J Austr. 2025 Feb 25;36(2):e70018. doi: 10.1002/hpja.70018 (PMC11862325; doi:10.1002/hpja.70018)

**Supplementary file 3.** Coding matrix for responses to quotes for Healthy Conversation Skills Training.

| **1st Response – Code** | **Description** | **Example** |
| --- | --- | --- |
| 0. Unable to code | The individual has not responded appropriately—i.e. they haven’t written what they would say, or they have reworded the statement rather than given a response. Or no response. | eg “I’d probably say something about exercising with their children”. Or “Find out if they know about our courses and give them a leaflet”. |
| 1. Telling / suggestions (giving information)  Sign-posting | Telling someone what to do; telling them something about themselves; giving information, including specific suggestions about what someone could try, or offering options. Might start with ‘what/how about’, ‘what if’ or ‘why don’t you’. | eg “Get a recipe book.” Or “It’s never too late to learn.” Or “Tell me about your day”. Or “Try running up and down the stairs everyday”. Or “How about walking to work”. |
| 2. In my/others experience | A statement with a specific example of how the responder deals with a situation, including agreeing with the statement, OR how others might deal with it. Could be viewed as “normalising” the behaviour. **Not** if they demonstrate own knowledge (ie telling, Code as 1), but when share own or others’ behaviour. | eg “I try to build it into my day.” Or “I find it difficult too.” Or “This is what I usually do…” Or “Other people feel like that & this has worked for them.” |
| 3. Reflection /empathy  (See Code 7) | A statement that indicates an understanding of the person, or their situation. Can be repeating back what they’ve said in different words, or clarifying understanding. (If precedes an ODQ, Code as 7). | eg “That must be difficult.” Or “Seems like you’d really like to do more exercise” |
| 4. Closed Question | A question with ‘yes’ or ‘no’ as possible answers | eg “Would you be interested in attending a workshop?” Or “Do you feel you have to go to a gym to exercise more?” |
| 5. Open Question (other) | A question that requires more than just a ‘yes’ or ‘no’ answer. May test knowledge, but does n**ot** support people to explore their current behaviour, verbalise the benefits or barriers to change, or to come up with their own solutions. Often starts with ‘why’, ‘when’, ‘who’, which or ‘where’. Can start with What/How if not exploratory/empowering. | eg “Why can’t you fit that into your life?” Or “When are quiet times for your family?” Or “Where do you do your shopping?” |
| 6. Open Discovery Question (But **not** 1st response) | The individual has provided an Open Discovery Question (see Code 7) somewhere in their response, but it is **not** the first thing written down. **Except** if 1st response is empathy (Code as 7). **NOT** if ODQ is lost in list of unrelated suggestions (code as 1). | eg “Would you like to learn how to cook and what to eat? How would it benefit your family’s life?” |
| 7. Open Discovery Question | A question that requires more than just a ‘yes’ or ‘no’ answer. Supports people to explore their current behaviour, verbalise the benefits or barriers to change, come up with their own solutions or make their own plan, ie is empowering. Begins with **‘what’** or **‘how’**. **NOT** ‘how about/what about’ or ‘what if’ (this is telling, Code as 1). Include when directly following empathy. | eg “What could you do to change this?” Or “How do you think you might find out?” |

**Supplementary file 4.** Figure. Impact of Healthy Conversation Skills training on staff use of open discovery style questions.

**Supplementary file 1.** Figure. Controlled trial study design timeline.

| October 2021 | |  | December  2021 | |  | March  2022 | | | | | | | |  | May  2022 | |
| --- | --- | --- | --- | --- | --- | --- | --- | --- | --- | --- | --- | --- | --- | --- | --- | --- |
|  |  |  |  |  |  |  |  |  |  |  |  |  |  |  |  |  |
| Baseline data collection^a^  n target=14  n control=13 | |  | Resource guides  delivered    n=20 | |  | Pre  HCS survey^b^  n=16 | |  | HCS training  n=16 | |  | Post  HCS survey^b^  n=16 | |  | Follow-up data collection^a^  n target=10  n control=8 | |
|  |  |  |  |  |  |  |  |  |  |  |  |  |  |  |  |  |
|  |  |  |  |  |  |  |  |  |  |  |  |  |  |  |  |  |
|  |  |  | Implementation support package^b^ | | | | | | | | | | |  |  |  |
|  |  |  |  |  |  |  |  |  |  |  |  |  |  |  |  |  |

^a^both Target and Control groups

^b^Target group only

**Supplementary file 2.** Figure. A description of HCS purpose, philosophy, skills and training delivery (43).


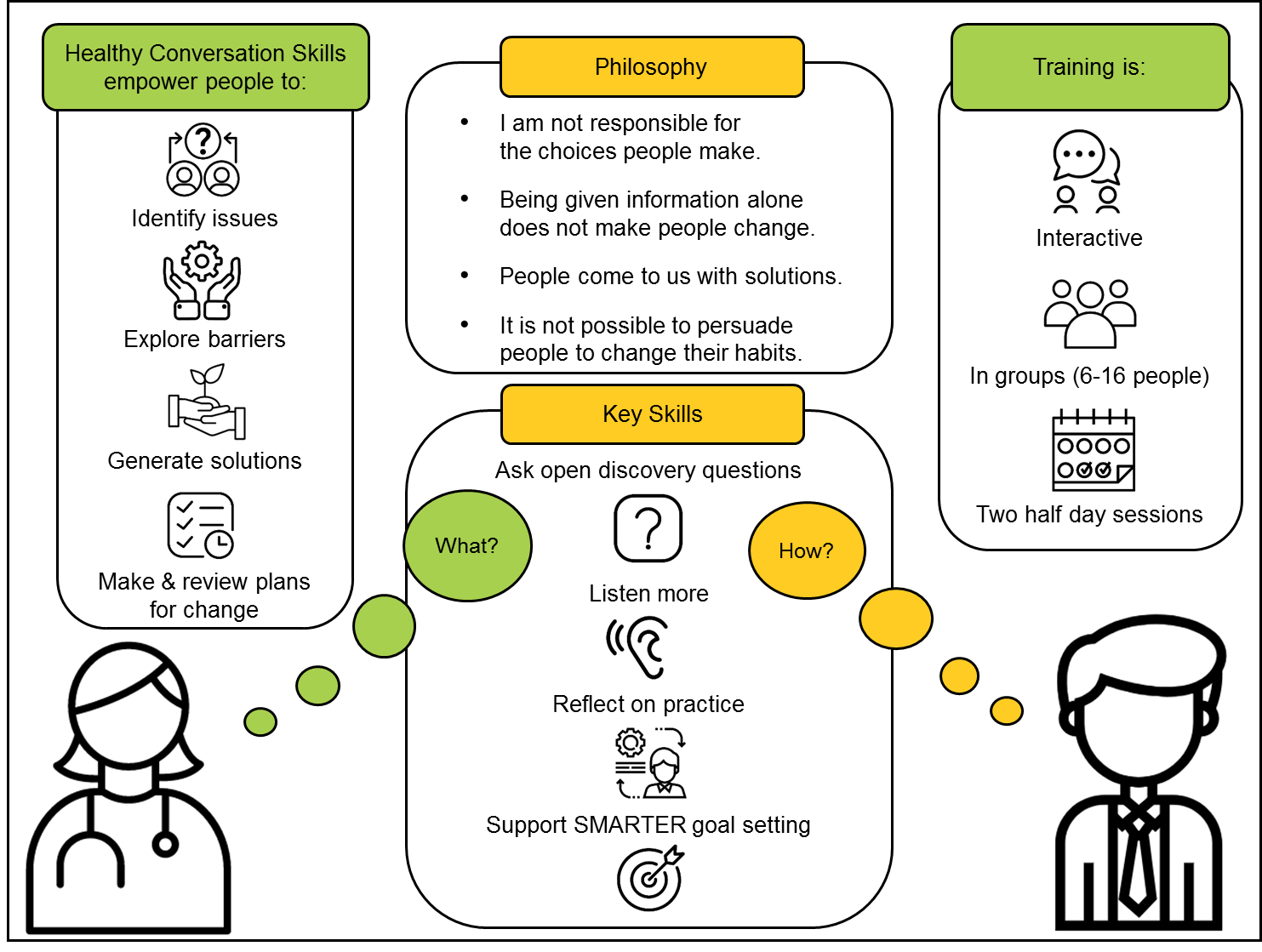

Supplement: Supplementary file 1 — Data S1: Supporting Information. [file HPJA-36-0-s001.docx]
